# Genomic competence among nurses: A spotlight on ethics

**DOI:** 10.1177/09697330251366594

**Published:** 2025-08-23

**Authors:** Mari Laaksonen, Eija Paavilainen, Anna-Maija Koivisto, Arja Halkoaho

**Affiliations:** Tampere University; 52917 Tampere University of Applied Sciences; Tampere University; Wellbeing Services County of South Ostrobothnia; Tampere University; 52917 Tampere University of Applied Sciences; University of Eastern Finland

**Keywords:** Genomics, competence, nurses, professional ethics, ethics of care

## Abstract

**Background:**

Globally, ethics is recognized as a critical component for ensuring equitable and sustainable genomic healthcare. However, prior research has largely overlooked the ethical aspects when assessing nurses’ genomic competence.

**Research aim:**

This study aimed to assess the genomic competence of nurses in Finland, with a specific focus on their perspectives regarding ethics in genomics.

**Research design:**

This was a cross-sectional study conducted among registered nurses in Finland.

**Participants and research context:**

The data were collected via an online survey between October 30 and December 31, 2023, using the Canadian Adaptation of the Genetics Genomics Nursing Practice Survey (GGNPS-CA), which evaluates attitudes, receptivity, confidence, competency, knowledge, social systems, and the decision adoption process in genomics with ethical dimensions. A total of 234 registered nurses participated.

**Ethical considerations:**

The study was ethically approved by the Ethics Committee of the Tampere Region, statement number 46/2023.

**Results:**

While 76.8% of nurses rated their self-assessed understanding of genomics as poor, their actual Knowledge Score was relatively good (mean 9.12/12, SD 1.44). In addition, nurses reported limited understanding of the ethical issues associated with genomics, particularly concerning equity. The majority (59.4%) believed it was very important for nurses to become more educated on ethical issues, while 28.6% considered it somewhat important.

**Conclusions:**

The findings suggest a strong perceived need among nurses for further education in both genomics and its ethical implications. The discrepancy between self-assessed and actual knowledge may reflect low confidence, which was additionally reported in the ethical issues. Low confidence is possibly influenced by the early stage of genomics integration into nursing practice.

## Introduction

Genomics has become an increasingly present part of healthcare in different sectors.^1^ It is a comprehensive field of science that requires competence among all healthcare professionals, including registered nurses, midwives, and public health nurses, regardless of their degree level or clinical setting.^2,3^ As the largest group of professionals in the health sector,^4^ nurses are well placed to help integrate genomics into routine health care, in order to maximize its benefits and opportunities effectively for society.^5^ This article examines the genomic competence of nurses in Finland, with a focus on their perspectives regarding ethics in genomics. Investigating this topic is crucial because sensitive genomics-informed nursing cannot be effectively provided without incorporating ethical considerations. This study is the first in Finland to explore genomic competence among practicing nurses. The research utilized the revised Canadian Adaptation of the Genetics and Genomics in Nursing Practice Survey (GGNPS-CA) instrument, which incorporates an important new dimension: ethics.^6^

## Background

Nurses’ preparedness to utilize genomics can be assessed by their attitude and competence, and these qualities are associated with their confidence to provide genomics-informed healthcare.^7^ However, even if professionals possess a high degree of self-assurance in their knowledge and capabilities, the integration of genomics into clinical practice may remain low,^8–10^ and vice versa, the widespread and developed applications of genomics do not necessarily ensure increased belief in one’s abilities. Carpenter–Clawson et al.^5^ reported a decline in nurses’ confidence levels despite the ongoing mainstreaming of genomics in England.

However, competence forms the foundation for confidence and implementation of knowledge. The genomic competence of registered nurses has been assessed globally in recent decades, using different instruments,^11,12^ and these studies have shown low-to-moderate competence and knowledge levels.^10,13–16^ There has also been little change in knowledge levels over the years, and the results have been consistent in different studies and countries.^16^ In terms of methodology, the genomic competence of nurses has been assessed in quantitative, qualitative, and mixed-method studies with cross-sectional and longitudinal approaches.^11,12^ In a few countries, namely, in USA, England, and Australia, researchers have carried out multiple studies. In contrast, in other countries such as Canada,^6^ China,^17^ Israel,^9^ Italy,^18^ Japan,^19^ Oman,^16^ and Turkey,^10^ only a few competence surveys have been conducted. In addition to the competence studies of registered nurses, there have also been several studies assessing the competence of undergraduate students,^15,19,20^ the development of genomic education,^21–23^ and effectiveness of education interventions for genomics-informed nursing.^24,25^ Nevertheless, the literature reveals a significant gap in evidence regarding the nursing workforce’s competence in genomics.

Ethics is acknowledged as an important part of genomics competence,^2,12,26^ and ethical elements are strongly present in care in the genomic era.^27,28^ In addition, some educational interventions have included ethics as a part of the objectives and contents of the courses conducted for bachelor and master level nurses.^19,25,29,30^ However, ethics is inconsistently integrated into competence assessment tools.^12^

Ethical considerations in the education of genomics and in procedures in practice are essential for nurses to work in an appropriately ethical way.^31^ Without the ability to understand ethical issues in genomics, nurses may encounter ethical distress and conflicts similar to those experienced by professionals in the field of genetic counselling.^32,33^ The sources of distress have been identified to involve other providers, family members, issues of responsibility, beliefs, and access to services.^33^ Although genomics is a relatively new innovation and its implementation remains incomplete and in developmental stages, the identification of ethical challenges related to genomics among nursing professionals dates back to the early 2000s. Informed decision-making, informed consent, privacy of information, and preventing discrimination were identified immediately as examples of ethical issues associated with genomics.^34^ In addition, some old and new competence and curricula guidelines incorporate genomic related ethical responsibilities and practice skills for nurses.^2,3,26^ The challenge of ethics in genomics is described in Williams and Dale’s^35^ study, where confidence in resolving ethical problems was identified as one of the weakest areas of competence among nurses.

The absence of ethical dimensions in many competence assessments has been criticized in recent reviews.^12,36^ Therefore, it is a positive move that this important element of genomics has been addressed further in the refining of one widely used instrument – The Genetics and Genomics in Nursing Practice Survey (GGNPS).^6^ This new ethics-enhanced Canadian Adaptation of the Genetics and Genomics in Nursing Practice Survey (GGNPS-CA) is used in this study to assess genomic competence and its ethical perspective. In particular, the lack of research on genomic competence among nursing professionals in Finland is a significant hindrance in integrating genomic knowledge into nursing skills and implementing genomic information into healthcare practices. This study aims to fill this gap by assessing the genomic competence and ethical awareness of nurses in Finland using the newly refined GGNPS-CA instrument, thereby supporting ethically sound healthcare delivery in the genomic era.

## Aims

This study aimed to assess the genomic competence of nurses in Finland, with a specific focus on their perspectives regarding ethics in genomics.

## Research design

This was a cross-sectional study conducted among registered nurses in Finland. The descriptive survey utilized the Canadian Adaptation of the Genetics and Genomics Nursing Practice Survey (GGNPS-CA), a modified version of the original Genetics and Genomics Nursing Practice Survey (GGNPS).^6^ Research Electronic Data Capture (REDCap 14.5.8) software was utilized to build and manage the online survey. The Finnish Nurses Association, The Finnish Association of Public Health Nurses, and The Federation of Finnish Midwives granted the research permission for the research.

### Participants and research context

The data were collected between October 30 and December 31, 2023. The three professional associations delivered invitations via newsletter, e-mail, or closed Facebook group. The invitation was projected to reach all members including students, nurses, educators, researchers, and other support members. Due to the absence of disaggregated membership registries, it is not feasible to provide a precise estimate of the potential target population. Eligible participants for the survey were Finnish-speaking nurses with a registered nursing degree. The study accepted nursing professionals from all healthcare sectors and levels of expertise, with a minimum requirement of holding a registered nurse degree. This also included public health nurses, midwives, and paramedics who have a registered nursing degree as a basis of their double degree in Finland. In total, 234 registered nurses participated in the study.

### Ethical considerations

Although the invitation and link to the survey were delivered through the nursing associations, it was not a requirement to be a member of any professional organization to participate in the survey. The privacy and voluntary participation of respondents were ensured. Responses were anonymous, and the data was stored in the Tampere University cloud service behind a two-step authentication security protocol. The study was ethically approved by the Ethics Committee of the Tampere Region, statement number 46/2023**.**

### Instrument

The instrument utilized in this study is the Canadian Adaptation of the Genetics and Genomics Nursing Practice Survey (GGNPS-CA). This is a modified version of the Genetics and Genomics Nursing Practice Survey (GGNPS). The GGNPS is used to assess the genomic knowledge and competence of nursing professionals.^13^ It includes domains of attitudes, receptivity, confidence, social system, adoption, and genomic knowledge based on the theory of knowledge dissemination by Rogers’ Diffusion of Innovation (DOI),^37^ previously explained in detail in Calzone et al.^8,13^ and Plavskin et al.^38^ surveys. In addition, GGNPS is aligned with the competency guidelines for nurses.^13^ It is available on the National Human Genome Research Institute^39^ website. Studies using this instrument have been conducted widely, for example, in the United States, China, and Turkey.^8,10,17^

The development work for GGNPS-CA was done in Canada, responding to needs for modifying the instrument.^6^ The new instrument retains the original framework while incorporating an important new dimension: ethics. The face and content validity of the revised instrument were confirmed with a sample of 10 nurses who were recruited through the professional networks of the research team.^6^ The development and structure of GGNPS-CA have been reported in a study of Limoges et al..^6^

The GGNPS-CA was chosen for this study based on conceptual literature and existing instruments. Literature reviews have demonstrated that many instruments used to assess genomic competence among nursing professionals often neglect the ethical dimensions,^12,36^ despite ethics being recognized as an important aspect of genomic competencies^2,3,40^ and included in several education intervention studies.^19,24,35,41^ GGNPS-CA emphasizes the overall competence of all nurses without focussing on any specific genetic area.^6^ Therefore, it serves as a crucial initial assessment in the absence of prior studies involving practicing nurses in Finland. Especially, this tool offers a valuable foundation for developing strategies to implement ethical genomics into Finnish nursing practice.

The GGNPS-CA was translated into Finnish by the first author of this study with support from the research teams expertise. Reviewers were consulted for specific terminology. The Finnish version of the instrument was evaluated using a sample of five nurses from diverse professional backgrounds. An official language reviewer translated the Finnish version back into English. This translated version and the content of the instrument were discussed with the GGNPS-CA developers, and the use of Finnish version of GGNPS-CA in this context was collectively approved.

In modifying process of GGNPS-CA, three direct ethical questions were added in addition to four questions that indirectly indicated ethics. In the Finnish version of GGNPS-CA, one question in the ethical section was omitted to better adapt to Finnish healthcare and society. Table 1 presents the ethical questions incorporated in GGNPS-CA and GGNPS.

**Table 1.** The ethical questions incorporated in GGNPS-CA and GGNPS.

GGNPS/GGNPS-CA	Individual questions
New direct ethical questions (GGNPS-CA)	Q5. How important do you think it is for nurses to become more educated about the ethical issues (e.g. informed decision-making) associated with genomics?
	Q17.3. Please rate your understanding of the equity issues associated with genomics.
	Q17.4. Please rate your understanding of the ethical issues associated with genomics.
New indirect ethical questions (GGNPS-CA)	Q2.5. Please indicate whether you think addressing the needs of underserved groups (those with health disparity) would be a potential advantage of integrating genetics of common health challenges into your practice.
	Q6.11. Please indicate how confident you are that you can provide culturally safe care that includes genomics.
	Q6.12. Please indicate how confident you are that you can collect and interpret the family history with sensitivity for diversity, heritage, and family composition.
	Q- Please indicate whether the statement is true, false, or you do not know: Access to the social determinants of health (e.g. education and literacy, income and social status, social supports and coping skills, and physical environments) impacts gene expression.^a^
Old indirect ethical question (GGNPS, GGNPS-CA)	Q3.3. Please indicate whether you think increasing patient anxiety about risk would be a potential disadvantage of integrating genetics of common health challenges into your practice.

^a^This GGNPS-CA question was not incorporated in the Finnish version.

The GGNPS and GGNPS-CA questionnaires include a Knowledge Score (KS) section of 12 questions.^6,13^ One question in KS was switched to another for the Finnish version of GGNPS-CA. The original included: ‘Extent to which family history supports clinical decisions (such as administering drugs prescribed)’. This was replaced by Knowledge Score Question (KSQ), KSQ10: ‘Do you think that genetic risk (e.g. as indicated by family history) has clinical relevance for mental health?’ The minimum and maximum scores remained the same. The KSQs of the Finnish version of GGNPS-CA are presented in Table 2.

**Table 2.** Knowledge Score Questions (KSQ) in Finnish version of GGNPS-CA and frequencies.

Knowledge score question (KSQ)	Correct answers for those respondents who answered all KSQ		Correct answers for all respondents separately in each KSQ	
	*n* = 216	%	n	%
KSQ1. A family history that includes only 1st degree relatives such as parents, siblings, and children should be taken for every new patient.	68	31.5	71 (*n* = 231)	30.7
KSQ2. A family history that includes second and 3rd degree relatives such as grandparents, aunts, uncles, and cousins should be taken for every new patient.	103	47.7	109 (*n* = 231)	47.2
KSQ3. Family history taking should be a key component of nursing care.	167	77.3	179 (*n* = 231)	77.5
KSQ4. There is a role for nurses in counselling patients about genetic risks.	174	80.6	188 (*n* = 231)	81.4
KSQ5. Do you think that genetic risk (e.g. as indicated by family history) has clinical relevance for breast cancer?	216	100	233 (*n* = 233)	100
KSQ6. Do you think that genetic risk (e.g. as indicated by family history) has clinical relevance for colon cancer?	216	100	227 (*n* = 227)	100
KSQ7. Do you think that genetic risk (e.g. as indicated by family history) has clinical relevance for coronary heart disease?	214	99.1	228 (*n* = 230)	99.1
KSQ8. Do you think that genetic risk (e.g. as indicated by family history) has clinical relevance for diabetes?	215	99.5	231 (*n* = 232)	99.6
KSQ9. Do you think that genetic risk (e.g. as indicated by family history) has clinical relevance for ovarian cancer?	210	97.2	224 (*n* = 230)	97.4
KSQ10. Do you think that genetic risk (e.g. as indicated by family history) has clinical relevance for mental health?^a^	214	99.1	228 (*n* = 230)	99.1
KSQ11. The DNA sequences of two randomly selected healthy individuals of the same sex are 90–95% identical.	72	33.3	79 (*n* = 233)	33.9
KSQ12. Most common health challenges such as diabetes and heart disease are caused by a single gene variant.	101	46.8	111 (*n* = 232)	47.8

^a^This KSQ differs from the original GGNPS.

The Finnish version of GGNPS-CA includes 18 main questions. Some of these questions contain sub-questions or statements. In total, 59 individual questions, sub-questions, or statements were included in the instrument divided into the same domains as GGNPS: attitudes, receptivity, confidence, genomic knowledge, social system, and adoption.^13^ The questionnaire included various types of questions, such as multiple choice, dichotomous, and Likert scale. Seven demographic questions were adjusted to fit the Finnish education degrees and healthcare context. Information concerning the channel through which the survey was found, age, gender, working role, highest level of nursing education, years in practice, and practice setting was collected.

### Statistical analyses

All collected data were exported from REDCap and imported into IBM Statistical Packages for the Social Sciences (SPSS) (29.0.1.0) for analysis. Categorical variables with sparse responses (1–3 responses in a category) were reclassified to improve analytical power. Specifically, three-level scales (excellent–good–poor) were collapsed into two-level categories (excellent/good–poor) for items Q5, Q17.1, Q17.2, Q17.3, and Q17.4.

The continuous Knowledge Score (KS) variable, ranging from 0 to 12, was calculated only for participants who completed all 12 knowledge items. Information about the individual questions of the KS is presented in Table 2.

Descriptive statistics, including frequencies, percentages, means, and standard deviations were computed. The normality of distributions was visually assessed to decide between employing parametric and non-parametric tests. For analysis of variance, the assumption of equality of variances was tested using Levene’s test. The KS and one demographic question (age) were quantitative and followed a normal distribution allowing analyses for T-test or One-way ANOVA, depending on the number of groups to compare. If the variances were not equal, Brown-Forsythe test statistics were used in One-way ANOVA. If the analysis of variance indicated differences in group means, Tukey’s pairwise comparison was used for further testing. Categorical associations were analyzed using the Chi-square test. A *p*-value of <0.05 was considered statistically significant.

## Results

### Demographics

The demographic features of the respondents are given in Table 3. Of the 234 participating nurses, the mean age was 43.93 years (SD = 11.0; range 24–70). Nearly all respondents were female (95.7%, *n* = 224). The majority (75.2%) held a Bachelor of Nursing Science (BNS), while 20.9% held a Master’s degree. 35.9% of the nurses had over 20 years of work experience in nursing, while 16.2% had 5 years or less. Most of the nurses (59.4%) described their current primary role in working life as a public health nurse, while 21.8% described working as a registered nurse. Midwives and paramedics constituted 4.2% of the respondents. 12.4% described their role as ‘other’. This group included roles such as occupational nurse, research nurse, coordinator, service manager, diabetes nurse, memory nurse, retiree, clinical specialist, or head nurse.

**Table 3.** Demographic features of the participants.

**Participants**	**n**	**%**
Demographics	231	
Missing	3	
Age (years)	216	92.3
Mean 43.93, SD 11.00		
Missing	18	7.7
Sex		
Male	1	0.4
Female	224	95.7
Prefer not to say	3	1.3
Missing	6	2.6
Highest nursing degree		
BNS – including:			
Old vocational education degree (degree ended in 1995)	52	22.2
Professional university degree (degree started in 1995)	124	53.0
MHS – including:			
Masters’ degree (Universities of Applied Sciences)	42	17.9
Master of health science (Universities)	7	3.0
PhD	1	0.4	
Something else	5	2.1	
Missing	3	1.3	
Primary role		
Registered nurse	51	21.8
Public health nurse	139	59.4
Midwife	9	3.8
Paramedic	1	0.4
Other	29	12.4
Student	1	0.4
Missing	4	1.7
Working experience (years) in nursing		
<1 year	4	1.7
1–5 years	34	14.5
6–10 years	41	17.5
11–15 years	40	17.1
16–20 years	28	12.0
Over 20 years	84	35.9
Missing	3	1.3

### Genomic knowledge

Of the respondents, 21% (*n* = 49) assessed their general genetic/genomic understanding in two-level variable as excellent/good, while 79 % (*n* = 184) assessed their understanding as poor. In comparison, the distribution of responses was more balanced regarding their understanding of the genetics of common health challenges such as diabetes and heart disease (excellent/good 43.8%, *n* = 102 and poor 56.2%, *n* = 131).

A total of 216 nurses (92.3%) answered all the questions in KS, and the mean KS was 9.12 (SD = 1.44). The minimum score received was 6/12 and the maximum 12/12. All respondents answered KSQ5 and KSQ6 correctly, acknowledging that genetic risk (e.g. as indicated by family history) has clinical relevance in breast and colon cancer. The most challenging questions were KSQ1 and KSQ11, with only 30.7% and 33.9% of respondents providing correct answers, respectively. KSQ1 addressed taking family history that includes only 1st degree relatives, and KSQ11 addressed the similarity of DNA between persons.

### Associations between variables and Knowledge Score

When a two-level classification (excellent/good – poor) of self-reported understanding of general genomics/genetics is compared to the KS, there is a statistically significant difference (*p* = .005) in means between the groups, according to the T-test (mean difference 0.68). Those who perceived their understanding as poor had a lower overall KS on average (*n* = 171, mean score 8.98) compared to those who perceived their understanding as good (*n* = 44, mean score 9.66). In addition, understanding of the genetics of common health challenges revealed a similar pattern (*p* = .001, mean difference 0.63). Those who perceived their understanding as poor had a lower overall KS on average (*n* = 120, mean score 8.83) compared to those who perceived their understanding as good (*n* = 95, mean score 9.46).

There was no statistically significant association between variables concerning the educational level of respondents (*p* = .326), the number of years working in nursing (*p* = .908), and whether their education included genetics (*p* = .099) in relation to the KS in this study. The associations between KS and different variables are presented in Table 4.

**Table 4.** Knowledge Score (KS) association to variables.

Variable	KS mean	SD	n	Test statistics^a^	Df^b^	*p*-value
Understanding of general genomics/genetics				2.814	213	0.005
Excellent/good	9.66	1.29	44			
Poor	8.98	1.46	171			
Understanding of the genetics of common health challenges				3.257	213	0.001
Excellent/good	9.46	1.37	95			
Poor	8.83	1.44	120			
Educational level				1.167	4; 209	0.326
Vocational education (BNS)	9.33	1.61	46			
University of applied science (BNS)	8.92	1.43	118			
Master studies in a university of applied sciences	9.33	1.36	39			
Master studies in a university	9.33	1.03	6			
Something else	9.60	0.55	5			
Number of years working in nursing				0.096	2; 211	0.908
Under 10 years	9.08	1.32	77			
10–20 years	9.18	1.59	61			
Over 20 years	9.09	1.46	76			
Education included genetics				2.360	2; 107.086^c^	0.099
Yes	9.47	1.63	62			
No	9.01	1.29	123			
Do not know	8.87	1.54	31			

^a^T test statistics for T-test, F test statistics for ANOVA.

^b^Degrees of freedom.

^c^Brown–Forsythe statistics.

### Ethical issues

Most nurses (59.4%) stated that it is very important for nurses to become more educated about the ethical issues (e.g. informed decision-making) associated with genomics, and 28.6% saw this as somewhat important. Only a minority felt that becoming more educated about ethics is not very important (3%) or not at all important (0.4%), while 8.6% of respondents felt neutral or did not know.

Majority (75%) reported their understanding of ethical issues associated with genomics to be at a poor level, while 25% stated it to be excellent/good. Regarding self-reported understanding of equity in genomics, only 15.2% of nurses rated their understanding as excellent/good, while 84.8% rated it as poor.

A Chi-Square test indicated the statistically significant association (*p* < .001) between understanding ethical issues and understanding equity. 56.1% of those who classified their understanding of ethics related to genomics as excellent/good also felt their understanding of equity in genomics to be at the same level. Conversely, nearly as many of the same respondents (43.9%) rated their comprehension of equity as poor. Vast majority of nurses (98.3%) who indicated that they understand ethics poorly, also perceived their comprehension of equity issues as weak.

Indirect questions of ethics included potential consequences and confidence in practicing ethical genomics. Addressing the needs of underserved groups divided nurses’ opinions. Half of the respondents (52.6%, *n* = 122) saw integration of genomics as beneficial for underserved groups. Most of the nurses (63.4%, *n* = 144) identified an increasing anxiety of patients due to genomics risk as a potential disadvantage. However, nurses lacked confidence. A significant majority (82.3%, *n* = 190) did not feel confident providing culturally safe genomic care. Additionally, 75.3% (*n* = 174) reported low confidence in collecting and interpreting family histories with sensitivity to diversity.

### Ethical issues and genomic knowledge

A statistically significant association between the importance of ethics education and KS (*p* = .003) was found. Based on Tukey’s pairwise comparisons, a significant mean difference of 1.22 (*p* = .004) was noticed between two groups. The respondents who rated ethics education as important scored higher on the KS (mean score 9.22) than those who stated neutral (mean score 8).

A T-test showed a statistically significant association between understanding of ethics and KS (*p* = .026, mean difference 0.51). The same was found between understanding of equity and KS (*p* = .001, mean difference 0.90). Consistently, nurses who expressed a poor understanding of ethics and equity achieved lower scores in the KS than those in the excellent/good group. The KS association to ethics is presented in Table 5.

**Table 5.** Knowledge Score (KS) association to ethical variables.

Variable	KS mean	SD	n	Test statistics^a^	Df^b^	*p*-value
Important to become more educated about ethics				5.940	2; 210	0.003^c^
Important	9.22	1.40	190			
Neutral	8.00	1.25	15			
Not important	8.50	1.93	8			
Understanding of ethical issues associated with genomics				2.241	212	0.026
Excellent/good	9.52	1.21	52			
Poor	9.01	1.48	162			
Understanding of equity in genomics				3.319	212	0.001
Excellent/good	9.88	1.41	32			
Poor	8.98	1.41	182			

^a^T test statistics for T-test, F test statistics for ANOVA.

b Degrees of freedom.

^c^The statistically significant difference between the two groups: Important and neutral (Tukey *p* = .004).

### Ethical issues and demographics

Working experience in nursing did not have a statistically significant association with either the understanding ethics (Chi-Square Test *p* = .372) or equity issues (Chi-Square Test *p* = .943). Additionally, the age of respondents was not statistically significantly associated with understanding ethics (T-test *p* = .148). The mean age for those nurses who stated an excellent/good understanding of ethics was 45.77. For those who assessed their understanding as poor, the mean age was 43.25. Similar results were seen when comparing age and understanding equity (T-test *p* = .184), where the mean age was 46.13 for an excellent/good understanding of equity and 43.31 for a poor understanding of equity. The results are described in Table 6.

**Table 6.** Association between working experience, age, and understanding ethical variables.

Demographics	Variable		Test statistics^a^	df^b^	*p*-value
	Understanding ethics in genomics				
	Excellent/good	Poor	1.978	2	0.372
Working experience	% (n)	% (n)			
Under 10 years	29.1 (23)	70.9 (56)			
10–20 years	19.1 (13)	80.9 (55)			
Over 20 years	25.3 (21)	74.7 (62)			
Age	Mean; SD (n)	Mean; SD (n)			
	45.77; 10.18 (53)	43.25; 11.22 (162)	1.452	213	0.148
	Understanding equity in genomics				
Working experience	Excellent/good	Poor	0.118	2	0.943
	% (n)	% (n)			
Under 10 years	13.9 (11)	86.1 (68)			
10–20 years	14.9 (10)	85.1 (57)			
Over 20 years	15.9 (13)	84.1 (69)			
Age	Mean; SD (n)	Mean; SD (n)			
	46.13; 9.70 (31)	43.31; 11.06 (182)	1.332	211	0.184

^a^T test statistics for T-test, χ2 test statistics for chi-square test.

^b^Degrees of freedom.

### Summary of the main results

• Nurses self-assessed their understanding of genomics as poor, although their mean KS was relatively good (9.12/12).
• Nurses who rated their understanding of genomics or ethics as excellent/good scored significantly higher on the KS than those who rated their understanding as poor.
• Nurses perceived their understanding of ethical issues and equity as poor, but they felt it was important to become more educated about ethical issues related to genomics
• Nurses did not feel confident providing culturally appropriate care in genomics

## Discussion

This study aimed to assess genomic competence and ethical issues associated with genomics among registered nurses in Finland. It showed that nurses critically self-evaluated their understanding of genomic knowledge compared to the KS test. The KS in this study was relatively good (mean = 9.12). A comparable score (9.36) was reported in a recent Turkish study,^9^ although the authors classified it as moderate. Similar levels were found in China (7.35) and in Canada (8.59).^6,17^ The threshold values were not mentioned in any previous studies. Given the absence of a universal standard for classification of KS, we implemented a 3-tier system based on percentage ranges^42^: Less than 50% (0–6) was considered as low, 51–75% (6.12–9.0) was considered as moderate, and 76–100% (9.12–12) was considered as good.^42^ However, these KS thresholds may not be directly comparable to previous studies, which did not specify categorization criteria, and one question (KSQ10) differed.

Most of the nurses in this study reported a limited understanding of ethics and equity in genomics, highlighting the importance of ethics education in this field. Ethics, in general, has long been regarded as an integral part of everyday nursing,^43,44^ and a vital part of quality healthcare.^45^ Ethical competence has been defined in terms of ethical awareness, moral judgement skills, the willingness to do good, and of character strength.^46^ In addition to these partly internal personal attributes, ethical competence is seen as developing within a social context.^47^ For nurses, this social context is the healthcare organization in which they work. Understanding healthcare professionals’ perceptions of ethics in genomic era enables nurse leaders and management to offer support that strengthens ethically robust genomics-informed practices. Ethics rounds in work settings could effectively support and train nurses by facilitating discussions on genetic related ethical issues. These interprofessional, low-hierarchy sessions allow professionals to engage in reflection^48,49^ providing them with self-confidence to resolve ethical questions independently later on. By using tools such as storytelling, ethics rounds can provide an effective educational platform for nurses^50^ to increase knowledge and confidence both needed for implementation of genomics.

This research contributes to understanding the current state of genomics and nursing in Finland. Internationally, it provides valuable benchmarks, highlighting the importance of ethically competent care for healthcare delivery in the genomic era. To date, ethics has been underrepresented in assessments of genomic competence. Our findings indicate that ethics should be integrated throughout the genomics implementation process, from curricula recommendations and educational tools to clinical practice, nursing leadership education, and interdisciplinary healthcare collaboration.

### Limitations

Some limitations are recognized in this study. The GGNPS-CA has not yet been validated, but the original GGNPS has been validated with test–retest reliability^51^ and validity evaluation.^39^ Due to changes in the utilized Finnish version of GGNPS-CA, direct comparison to other surveys using the original GGNPS and GGNPS-CA requires precision. However, instruments should always evolve in response to changing information, emerging needs, and different societal contexts. The added ethical questions ensure that all aspects of genomic competence are considered in the future.

The authors acknowledge the potential for response bias, which may arise from the likelihood that nurses with a greater interest in genomics were more inclined to respond. Additionally, the distribution of nursing professional groups responded the survey does not fully correspond to the workforce composition in Finland. The largest group completing the survey was public health nurses, whereas the largest group of working professionals are registered nurses. However, the results remain generalizable because, in Finland, public health nurses are also qualified and licensed to work as registered nurses.

Because the study was not limited only to hospital or outpatient settings, the primary field of practice of the respondents expanded to encompass the entire healthcare sector. The majority of nurses reported working in an outpatient setting. Thus, the survey results reflect better outpatient nurses’ perceptions and competence. However, this study provides essential information about the state of genomics-informed nursing, being the first of its kind in Finland. The results may be utilized to develop educational interventions, frameworks, guidelines, and to raise awareness of ethical genomics among all nurses. A broad and interprofessional approach is considered advantageous in education,^52^ and this study offers the perceptions of various nursing professionals. Therefore, the comprehensive results are valuable for establishing general genomics training, including ethics, for all nurses, regardless of discipline. Although the study was conducted in only one country, it promotes ethical discussion globally.

## Conclusions

Nurses self-evaluated their understanding of genomics as poor, although the average score in KS indicated a good level of knowledge. Nurses who perceived their understanding to be poor tended to receive lower scores in KS, while those who stated they had a good understanding performed better. Thus, the self-evaluated question and the scored knowledge test complemented each other. The results indicate that nurses have a relatively accurate awareness of their genomic competence although they may underestimate their abilities.

A significant majority reported poor understanding of ethics and equity in genomics. Nurses did not feel confident in ethical issues and emphasized the need for additional training for nursing professionals. Low confidence may be influenced by the early stage of genomics integration into nursing practice in Finland.

As the first study of its kind in Finland, this research provides a foundation for creating a national genomics-informed nursing strategy. By addressing both genomic knowledge and its ethical dimensions, nursing education can better prepare practitioners for the growing demands of genomics-informed care. Future research should aim to validate ethical assessment instruments and evaluate the effectiveness of educational interventions in both genomics and its ethical implications.
